# Rosai-Dorfman Disease With Features of IgG4-Related Sclerosing Disease: A Case Report

**DOI:** 10.7759/cureus.27704

**Published:** 2022-08-05

**Authors:** Nasser M AlMadan, Mohammed K Alwhabi, Assem S Assem, Hatem Khoja

**Affiliations:** 1 Oral Medicine, Prince Sultan Military Medical City, Riyadh, SAU; 2 Central Laboratory and Blood Bank, Prince Sultan Military Medical City, Riyadh, SAU; 3 Pathology, Prince Sultan Military Medical City, Riyadh, SAU; 4 Anatomic Pathology, King Faisal Specialist Hospital and Research Centre, Riyadh, SAU

**Keywords:** lymphocyte, cell proliferation, immunoglobulin g4-related disease, sinus histiocytosis, histiocytes

## Abstract

Rosai-Dorfman disease (RDD) is a rare condition characterized by the proliferation of non-Langerhans cell histiocytes that are associated with phagocytosed lymphocytes (emperipolesis). Clinically, it is classified into nodal, extra-nodal, neoplasia-associated RDD, and immune-related. Here, we present a case of a 65-year-old female who presented with facial pain following a dental procedure with no focal neurologic deficit. The MRI of the head and neck showed a well-defined lobulated soft tissue lesion with homogenous enhancement over the left cheek. Excision of the lesion was done, and the histopathological study reported extra-nodal RDD with features of IgG4-related sclerosing disease. The patient had no recurrence over the two years from the date of diagnosis.

## Introduction

Sinus histiocytosis or Rosai-Dorfman disease (RDD) is a non-malignant health condition. It is usually presented as a bilateral painless massive cervical lymphadenopathy associated with fever, night sweats, fatigue, and weight loss [1]. From the context of histopathology, It is characterized by histiocytic proliferation with hypochromic nuclei and abundant pale cytoplasm with phagocytosed lymphocytes (emperipolesis) and background of polyclonal plasma cells [2].

RDD is a heterogeneous group of disorders with a variety of clinical and radiographic features. There are two main clinical types of RDD: the first one occurs in the lymph node (nodal), and the second is the extra-nodal which is considered the most frequent variant located in the skin, nasal cavity, soft tissue, and orbit [1,3].

The immunohistochemically study usually reveals S100 and CD68 positive cells but negative for CD1a or CD207 staining [3]. Moreover, RDD frequently contains abundant IgG4-positive plasma cells, and there is a recommendation regarding the necessitating evaluation of the ratio of IgG4+ plasma cells to IgG+ cells and the numbers of FOX3+ regulatory T-cells in order to exclude IgG4-related disease (IgG4RD) [4].

RDD with features of IgG4RD is seen in various age groups with a mean age of 30 years (1-69 years) in nodal and extra-nodal distribution. The cervical lymph node is the most involved site in some cases of multiple organ systems involvement [5,6].

In the Saudi medical literature, there is a need to explore this rare case and share the clinical experience for the purposes of early identification and proper clinical management. The current study presents a rare case of RDD in an old female and discusses the clinical and histological features in conjunction with the literature review.

## Case presentation

A 65-year-old female patient came to our clinic complaining of left facial pain that started after a dental procedure. Her pain was constant, electric-like, and not associated with neurological deficit. There was no history of seizure, fever, or change in taste or vision. She has a medical history of diabetes mellitus type 2, hypertension, and hypothyroidism. There were no symptoms of IgG4-related systemic diseases such as diffuse pain of joints, and tendons, with associated fatigue.

**Figure 1:**
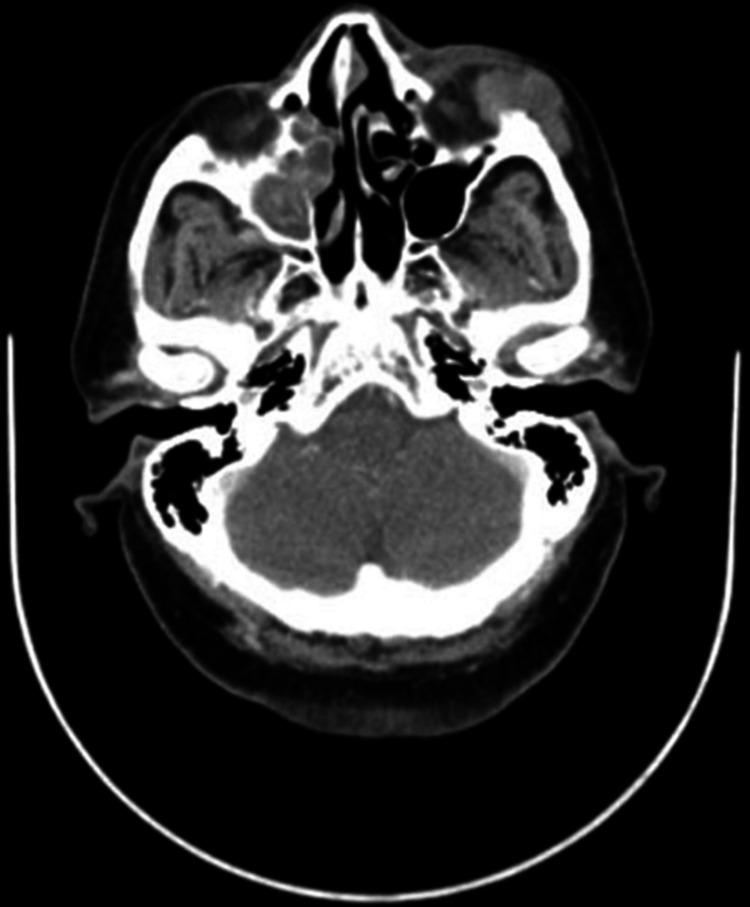
CT axial view of the head shows the subcutaneous mass overlying the left maxillary sinus

**Figure 2:**
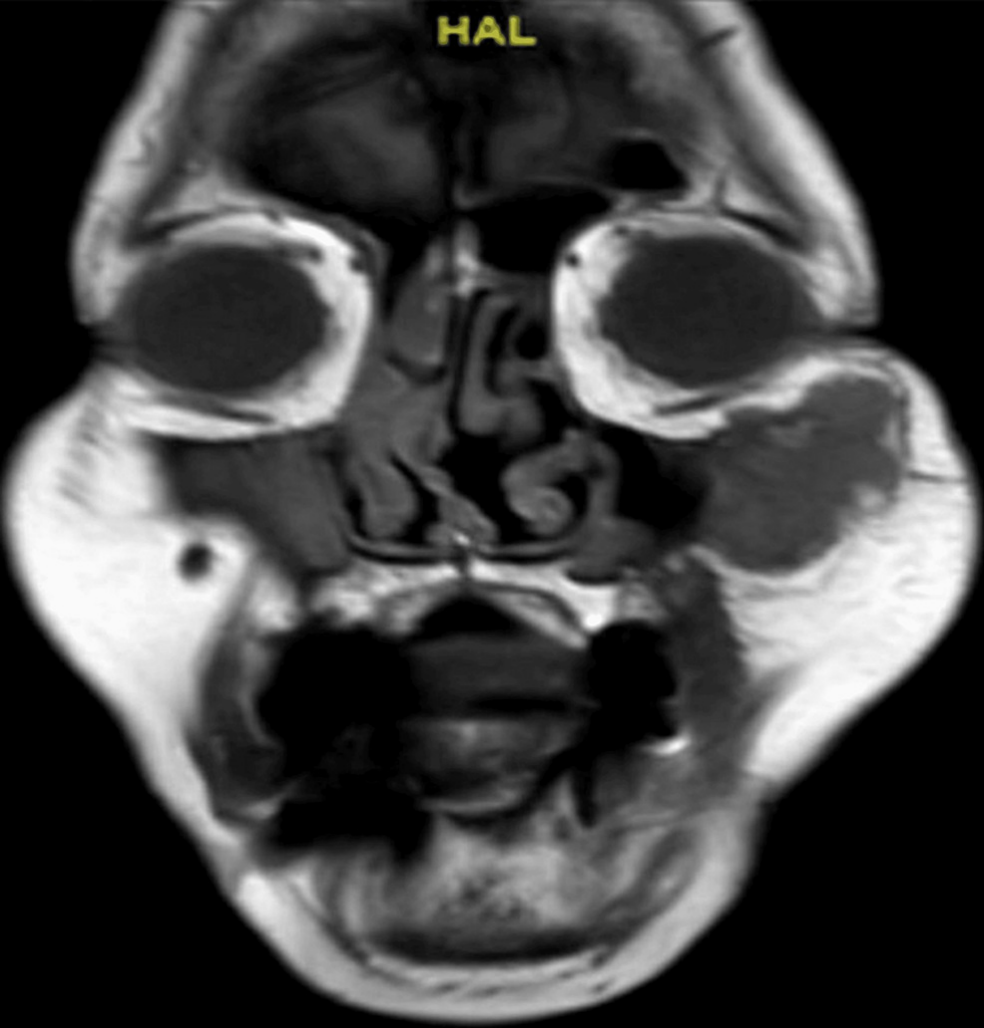
Head MRI shows well-defined lobulated soft tissue mass noted at the anterior left cheek subcutaneous area, with low to iso signal intensity

Clinical examination revealed unremarkable neurological work up aside from decreased sensation over the left cheek. Initial CT scan (Figure 1) showed a subcutaneous heterogeneous soft tissue mass measured 3.6 by 2.5 cm in maximum diameter and located overlying the left maxillary antrum extending over the inferior orbital rim with a lack of significant enhancement. Due to examining the soft tissues, the MRI (Figure 2) showed a well-defined soft tissue mass in the subcutaneous area of the anterior left cheek with low to iso-signal intensity in T1 with homogenous post-contrast enhancement.

**Figure 3:**
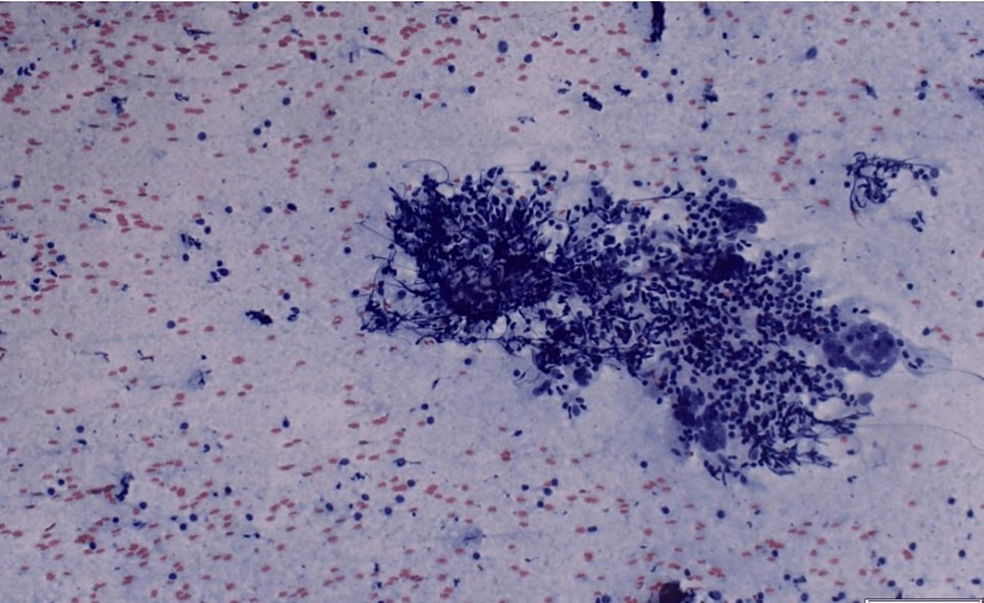
FNA shows a cluster of epithelioid cells in the background of inflammatory cells FNA - Fine Needle Aspiration

For further assessment, the ultrasound guide fine needle aspiration (FNA) was suggested, and it showed atypical epithelial cells admixed with chronic inflammatory cells (Figure 3). Incisional biopsy demonstrated fibro-inflammatory and histiocytic lesion with polyclonal lymphoid and plasma cell proliferation.

**Figure 4:**
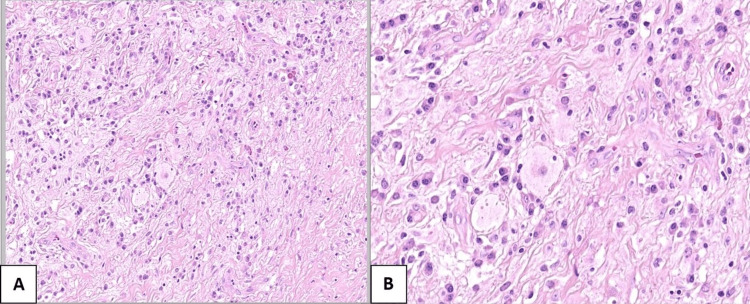
Intermediate-power (A) and high-power (B) histology slides demonstrating many histiocytes associated with plasma cells and lymphocytes

However, our patient underwent excisional surgery, and the sample was sent to the pathology department. The pathologists reported (Figures 4A, 4B) stromal fibrosis with a storiform pattern of multiple nodules showing mixed inflammatory cells of lymphocytes, plasma cells, and histiocytes.

**Figure 5:**
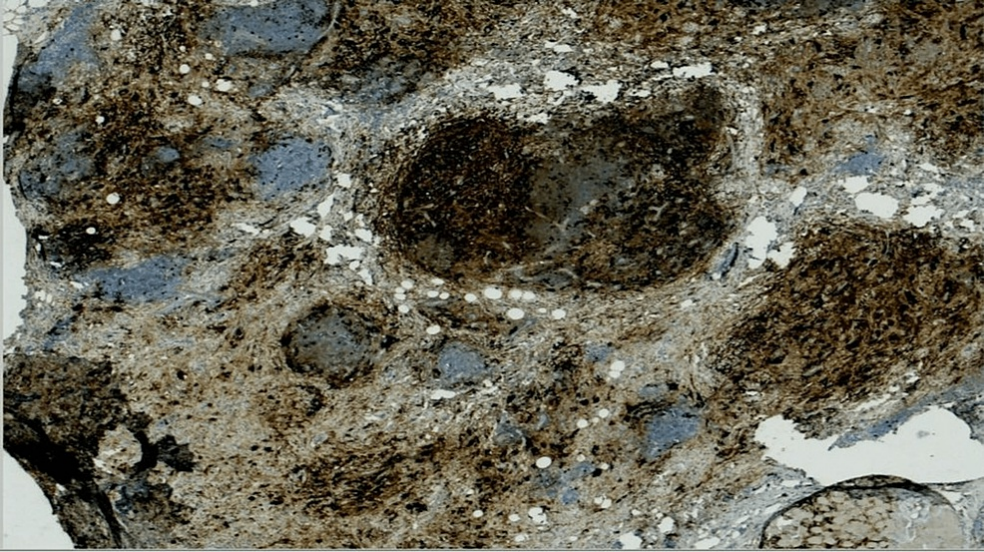
Immunostaining for IgG4 shows many IgG4+ plasma cells in the stroma

Lastly, the lesion was diagnosed as extra-nodal RDD with features of IgG4-related sclerosing disease (Figure 5). The patient was on regular follow-up and recorded no recurrence for two years following the excision.

## Discussion

RDD is an uncommon disease affecting the hematopoietic system. The typical clinical feature is painless cervical lymphadenopathy which is usually associated with general symptoms of fever, pain, and weight loss. The extra-nodal type is frequently located in the skin, nasal cavity, soft tissue, and orbit. The present case was visiting the clinic for the main complaint of pain due to extra-nodal RDD in the left maxilla.

A previous study [7] reviewed the literature over the last 40 years and found only eight cases of RDD located in the maxillary bones of 6 females and two males. This may highlight that extra-nodal involvement of RDD in maxillary bone affects female patients more than males, and it comes in line with our present case.

Histological and immunohistochemical studies are the gold standard for RDD diagnosis. There is a debate about the possible association between RDD and IgG4RD due to the similar histopathological appearance of IgG4+ plasma cells [8]. However, the pathogenic histopathological feature for IgG4RD is the presence of mass-forming lesions that form from the lymphoplasmacytic infiltrate, storiform fibrosis, and obliterative phlebitis [5,8]. Our patient demonstrated such immunohistochemical features, including positivity for CD68, S-100, and CD3, and was negative for CD30 and CD1a.

In the context of treatment, there are several options for treatment that range from doing nothing to undergoing excisional surgery. Researches indicated that a half of patient with RDD did not require any treatment and other patient had steroids, antibiotics, antifungal, chemotherapy, surgery, or radiation [9]. However, the combined treatment was reported in previous studies [9,10]. The option of treatment in the present case was excisional surgery that helped for confirming the diagnosis, and patient after that was on strict follow-up. After a two-year of follow-up, the patient was asymptomatic, and her health condition was considered to be under control, necessitating no more treatment.

## Conclusions

RDD that affects the maxillary bone is exceedingly uncommon and needs to be identified and differentiated from other diseases by performing histological studies. Strict follow-up after the treatment is essential to monitor the possibility of recurrence.
